# Urological cancer patients receiving treatment during COVID-19: a single-centre perspective

**DOI:** 10.1038/s41416-021-01263-7

**Published:** 2021-02-08

**Authors:** Sophie Therese Williams, Salma El Badri, Syed Anwer Hussain

**Affiliations:** 1 Department of Oncology and Metabolism, University of Sheffield, Broomhall, Sheffield UK; 2 Weston Park Hospital, Broomhall, Sheffield UK

**Keywords:** Cancer epidemiology, Bladder cancer, Prostate cancer

## Abstract

**Background:**

Active cancer, immunosuppressive treatments and immunotherapies have been reported to increase cancer patients’ risk of developing severe COVID-19 infection. For patients and clinicians, treatment risk must be weighed against disease progression.

**Methods:**

This retrospective case series surveys urological cancer patients who made informed decisions to continue anticancer treatment (ACT) at one centre from March to June 2020.

**Results:**

Sixty-one patients (44 bladder, 10 prostate, 7 upper urinary tract cancers) received 195 cycles of ACT (99 chemotherapy, 59 immunotherapy, 37 as part of ongoing clinical trials), with a range of indications: 43 palliative, 10 neoadjuvant, 8 adjuvant. One patient tested positive for COVID-19 but experienced only mild symptoms. Fourteen patients interrupted treatment outside of their schedule, seven of these due to potential COVID-19 associated risk. ACT supportive steroids were not associated with higher rates of COVID-19.

**Conclusions:**

This single-centre series reports that ACT administration did not result in an apparent excess in symptomatic COVID-19 infections.

## Background

The evolving COVID-19 pandemic, caused by Severe Acute Respiratory Syndrome Coronavirus 2 (SARS-CoV-2), is a huge concern for oncologists and cancer patients. Early reports suggested active cancer, immunosuppressive treatments and immunotherapy (IO) increased patients’ risk of developing severe COVID-19 infection.^1,2^ Early discussions of risk also focused on potential side effects of IO, requiring immunosuppressive agents such as steroids. Concern was raised regarding service provision, hospital attendance and infection exposure during treatment.^3^ NICE published guidance on how best to prioritise treatments, in the event that treatments needed to be rationalised, based on likelihood of cure, or extending life beyond 12 months.^4^

In urothelial cancers, there is an established evidence base for both palliative chemotherapy and immunotherapy,^5,6^ and advanced disease can progress quickly without palliative chemotherapy.^7^ For patients and clinicians, the risks from COVID-19 infection have to be weighed against the personalised risks of disease progression without treatment.

## Methods

Retrospective data were collected for all patients who attended for ACT for urological cancer between 1 March 2020 and 30 June 2020, under the care of one consultant at one centre (adjoined to infectious diseases, ICU and critical care services). Data were collected by review of electronic chemotherapy prescriptions, written and electronic clinical notes in the second week of July. Data included: demographic, diagnosis, ACT regimen, including presence or absence of supportive steroid, any reason for ACT interruption, emergency admissions. Biochemical results, oxygen requirement, NEWS-2 score on admission and COVID status were recorded if admitted to our centre or regional hospitals within South Yorkshire. All patients were screened for COVID-19 symptoms and contacts on entry to the hospital and on arrival at the chemotherapy day case unit. All patients receiving ACT were advised to follow the government’s advice on shielding. The potential risks of COVID-19 were discussed with all patients, including the potential implications should they require hospitalisation.

## Results

Sixty-one patients (44 bladder, 10 prostate and 7 upper urinary tract primary cancers) received a total of 195 cycles of anticancer therapy: 99 cycles of chemotherapy, 59 immunotherapy and a further 37 cycles of treatment as part of ongoing clinical trials (see Table 1). Median age was 71 (47–87) and included 41 men (67%). All patients had an ECOG performance status of 0 (41, 67%) or 1 (20, 33%) at clinical review. Metastatic disease was present in 45 patients (74%): 36 had visceral metastases and 9 had lymph-node only metastatic disease. There was a range of indications for treatment: 43 palliative, 10 neoadjuvant and 8 adjuvant. Across all patients, 48 had at least one comorbidity (79%), with hypertension being the most prevalent (21, 34%), followed by hearing impairment (9, 15%) and raised cholesterol (8, 13%). With regards to concomitant use of steroids, 10 patients received a 3-day course of dexamethasone with 21 days of prednisolone (as per standard Docetaxel chemotherapy regimen), while 29 patients received a short course (3 days) of dexamethasone as an antiemetic with a number of chemotherapy regimens (see Table 1). Two patients received an extended course of high dose prednisolone (>21 days) for immunotherapy-associated hepatitis (Grade 2 and Grade 3), neither of whom contracted COVID-19. The patient with Grade 3 hepatotoxicity was rechallenged with Atezolizumab in May 2020 without any adverse event.

**Table 1:** Patient anticancer therapy (ACT) regimens March 1 to June 30, 2020.

Type	Treatment	Steroid	Standard duration (days)	Cycles	Patients
IO	Atezoliazumab	—	—	51	12
IO	Pembrolizumab	—	—	8	2
CHEMO	Carbo/Etoposide	DEX	3	7	2
CHEMO	Cis1/Gem1 + 8	DEX	3	17	6
CHEMO	Cis1 + 8/Gem1 + 8	DEX	3	33	15^a^
CHEMO	Docetaxel	DEX/PRED	3/21	28	10
CHEMO	Gemc/Carbo	DEX	3	7	3
CHEMO	Paclitaxel	DEX	3	7	2^a^
TRIAL	POTOMAC (Durvalumab)	—	—	18	4
TRIAL	JAVELIN (Avelumab)	—	—	4	1
TRIAL	NIAGARA (Cis/Gem)	—	—	5	2
TRIAL	STRONG (Durvalumab)	—	—	4	1
TRIAL	ASTELLAS (Enfortumab)	—	—	4	1
TRIAL	ASTELLAS (Paclitaxel)	DEX	3	2	1
Total	**—**	**—**	**—**	195	61^a^

Duration of steroid in days. Patients receiving treatment on the ASTELLAS trial are presented by treatment type.

*DEX* dexamethasone, *PRED* prednisolone, *IO* immunotherapy, *CHEMO* chemotherapy, *TRIAL* patients receiving treatment on treatment on existing clinical trials.

^a^One patient received two treatments (Paclitaxel followed by Cis1 + 8/Gem1 + 8), making a total of 61 patients.

One patient tested COVID-19 positive while admitted for treatment-associated complications: they experienced mild COVID-19 symptoms that were managed on a ward and did not require oxygen therapy. Ten other patients required emergency admission to hospital, not related to COVID-19. Three patients had elective admissions: two had radical cystectomy following neoadjuvant chemotherapy and one had a nephrostomy insertion. All patients admitted, except two, were tested for COVID-19 with only one positive result as described above. Only symptomatic patients were tested for COVID-19 at the start of the pandemic, but the hospital policy changed in April 2020 to testing all admissions.

Fourteen patients interrupted treatment outside of their planned schedule. Seven were due to individual risk and COVID-19 (including the COVID-19 positive patient reported above), while the additional seven were due to non-COVID causes including: disease progression,^3^ immunotherapy-associated hepatitis,^2^ neutropenic sepsis^1^ and ischaemic stroke.^1^ This patient, with T2 N0 M0 bladder cancer, developed an ischaemic stroke after three cycles of neoadjuvant chemotherapy and was referred for radical, surgical treatment omitting the final cycle of chemotherapy.

## Discussion

This retrospective case series reports on the outcomes of patients who received systemic ACT during the peak of the first wave of COVID-19 infections in the UK. The demographics of these patients reflect a typical urological cancer cohort fit for systemic treatment: median age of 71, predominantly male with a performance status of 0 or 1 and relatively few comorbidities. In a 3-month comparable pre-COVID period, Sept 1 to Dec 31, 2019, 59 patients received 166 cycles of ACT, showing that clinical practice during our observation period was not dramatically reduced.

Only one patient tested positive for COVID-19 on admission to hospital for other reasons. This in part reflects the dedication of patients to following social distancing and shielding advice, but needs to be considered alongside the regional infection rate (R0), which in Sheffield has been reported as 0.983, the third highest local authority infection rate in the country as of the last week of June.^8^ It also demonstrates that of the 195 cycles given, and necessary treatment-associated visits, social distancing and screening questions were effective in preventing COVID-19 transmission. No patients required ICU level support or had died due to COVID-19 as of July 15, 2020, but it is important to note that ICU and critical care services were stretched throughout our study period. Our results are in keeping with the findings of Lee et al., who, after adjusting for age, sex and comorbidities, found no significant association between chemotherapy or immunotherapy in the past 4 weeks and mortality from COVID-19.^9^

Guidelines have advised avoiding the use of IO during COVID-19 because of the proposed risk of severe disease in immunosuppression resulting from IO-associated toxicity treatment with steroids.^4,10^ However, local and national observational studies have now refuted this mechanism.^9,11^ In fact, dexamethasone is now evidenced for treatment of COVID-19 infected patients requiring supplemental oxygen and or ventilation.^12^ For patients treated at our centre, IO treatment and IO toxicity treated with steroids did not result in COVID-19 infection.

It should be emphasised that this is retrospective data from a single institution, by a single consultant and only details the results for a narrow spectrum of cancers. We only captured patients who received treatment, not all patients who may have been considered for systemic treatment, including patients who decided to proceed with radical surgical treatment without neoadjuvant chemotherapy, or patients who declined adjuvant chemotherapy after surgery. We also may not have captured patients who have been COVID-19 positive in the community, but who did not seek medical attention or report symptoms at clinic or chemo-suite attendance.

## Conclusion

This single-centre, retrospective case series shows that patients were keen to continue treatment where possible, that ACT could be administered in a safe environment that did not result in an apparent excess in symptomatic COVID-19 infections. The administration of supportive steroids did not result in higher rates of infection, or severe disease, and that rates of chemotherapy and immunotherapy-associated complications were no higher than in pre-COVID-19 times. Our data demonstrates that treatment with systemic chemotherapy, immune checkpoint inhibitors and the use of steroids for toxicity management may be possible during the current COVID-19 pandemic, as now supported by Lee et al. and the RECOVERY trials.^9,12^ As the NHS begins routine clinical work again, patients need to make informed decisions after careful discussion with their oncologists about their treatments, and require careful review of risks from ACT, COVID-19 and disease progression.

## Data Availability

Data are available by request to the corresponding author.
